# Blocking protein phosphatase 2A signaling prevents endothelial-to-mesenchymal transition and renal fibrosis: a peptide-based drug therapy

**DOI:** 10.1038/srep19821

**Published:** 2016-01-25

**Authors:** Yuanjun Deng, Yanyan Guo, Ping Liu, Rui Zeng, Yong Ning, Guangchang Pei, Yueqiang Li, Meixue Chen, Shuiming Guo, Xiaoqing Li, Min Han, Gang Xu

**Affiliations:** 1 Division of Nephrology, Department of Internal Medicine, Tongji Hospital, Tongji Medical College, Huazhong University of Science and Technology, Wuhan, Hubei, People’s Republic of China

## Abstract

Endothelial-to-mesenchymal transition (EndMT) contributes to the emergence of fibroblasts and plays a significant role in renal interstitial fibrosis. Protein phosphatase 2A (PP2A) is a major serine/threonine protein phosphatase in eukaryotic cells and regulates many signaling pathways. However, the significance of PP2A in EndMT is poorly understood. In present study, the role of PP2A in EndMT was evaluated. We demonstrated that PP2A activated in endothelial cells (EC) during their EndMT phenotype acquisition and in the mouse model of obstructive nephropathy (i.e., UUO). Inhibition of PP2A activity by its specific inhibitor prevented EC undergoing EndMT. Importantly, PP2A activation was dependent on tyrosine nitration at 127 in the catalytic subunit of PP2A (PP2Ac). Our renal-protective strategy was to block tyrosine127 nitration to inhibit PP2A activation by using a mimic peptide derived from PP2Ac conjugating a cell penetrating peptide (CPP: TAT), termed TAT-Y127WT. Pretreatment withTAT-Y127WT was able to prevent TGF-β1-induced EndMT. Administration of the peptide to UUO mice significantly ameliorated renal EndMT level, with preserved density of peritubular capillaries and reduction in extracellular matrix deposition. Taken together, these results suggest that inhibiting PP2Ac nitration using a mimic peptide is a potential preventive strategy for EndMT in renal fibrosis.

Progressive tubulointerstitial fibrosis is the final common pathway for all kidney diseases leading to chronic renal failure^1^. Accumulating evidence suggests that the vascular component of the kidney is the best indicator of the progression of renal dysfunction^2^,^3^. Renal microvascular endothelial cells serve as “conductors” in the kidney and take center stage in renal fibrosis^4^. Endothelial cells (ECs), which provide a barrier between the bloodstream and tissues, are the primary target of various stimulations. In response to sustained injury, endothelial cells detach from peritubular capillaries, migrate to the interstitial space and become scar-forming myofibroblasts, a process called endothelial-to-mesenchymal transition (EndMT)^5^. Recent studies have demonstrated the involvement of EndMT in various types of fibrosis^6^,^7^. Moreover, EndMT has been shown to be pivotal in the development of chronic kidney disease^8^.

The vast majority of biological processes, including renal fibrosis, are regulated by the balance between the activities of kinases and phosphatases^9^. The significance of kinases has been intensively discussed in kidney disease for decades, however, the roles of phosphatases remain poorly understood. Protein phosphatase 2A (PP2A) is a ubiquitously expressed protein serine/threonine phosphatase that accounts for a large fraction of phosphatase activity in eukaryotic cells^10^. As a heterotrimer, PP2A is composed of three subunits: a catalytic C subunit (PP2Ac), a structural A subunit (PP2Aa) and a regulatory B-type subunit (PP2Ab). PP2Ac has two isoforms: α and β. However, PP2Ac_α_ is about 10 times more abundant than PP2Ac_β_^11^. Several lines of evidence suggest that PP2A is associated with endothelial instability^12^. PP2A substrates include membrane or cytoskeletal proteins, and abnormalities of PP2A lead to disturbances in these proteins and result in endothelial dysfuncton^13^,^14^. PP2A directly dephosphorylated occludin, a transmembrane protein of tight junctions and led to their disassembly^15^. Inhibition of PP2A by okadaic acid abolished its effect on thrombin- or nocodazole-induced changes in EC cytoskeleton, which indicates a critical role of PP2A activity in EC cytoskeletal maintenance^16^. Therefore, we hypothesized that PP2A activation would be involved in the induction of EndMT and could be a promising therapeutic target in renal fibrosis.

## Results

### In patients with primary and secondary nephropathy, the mesenchymal marker α-smooth muscle actin (α-SMA) is expressed in the endothelium

**Figure 1:**
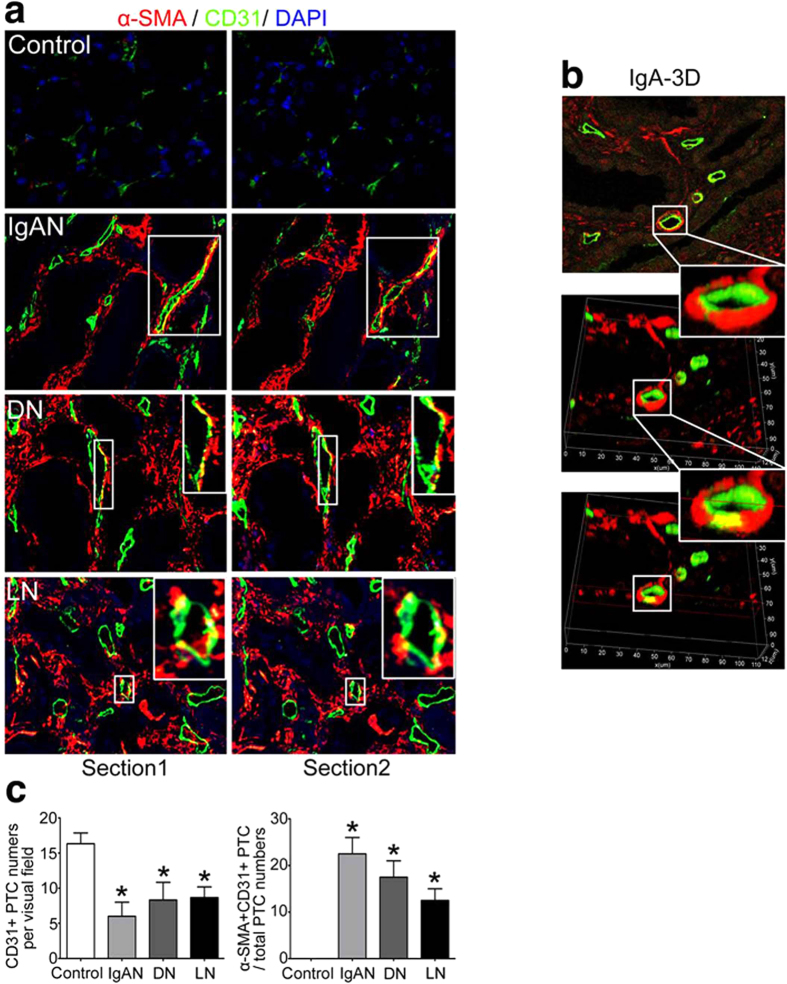
EndMT occurs in patients with primary and secondary nephropathy. (**a**) Co-localization of α-SMA (red) and CD31(green) on serial sections of kidney from patients with renal fibrosis. These were representative photomicrographs from patients with IgAN, DN and LN compared with controls at a magnification of ×800 (n = 8, 5 hpf per mouse, 40 hpf per group). (**b**) Representative 3D reconstructed images from patients with IgA illustrated microvascular endothelial cells underwent endothelial-to-mesenchymal transition. (**c**) The bar graphs summarized the average of CD31+ PTC numbers per visual field and the percentage of α-SMA+CD31+ peritubular capillary (PTC) numbers in total CD31+ PTC numbers in each group. *P < 0.05. Bars represent means ± sd.

To investigate the contribution of EndMT to the development of renal interstitial fibrosis, renal biopsy tissues from adult patients with primary nephropathy (e.g., IgA nephropathy, IgAN^17^) and secondary nephropathy (e.g., diabetic nephropathy, DN; lupus nephritis, LN) were analyzed. Sequential kidney sections were used to confirm the appearance of EndMT. Using double labeling of tissue for endothelial (CD31) and fibroblast marker (α-SMA), we demonstrated that α-SMA+/CD31+ in the interstitium was highly congruent on sequential sections (Fig. 1a). To further prove EndMT, 3D confocal laser-scanning microscopes (3D-CLSM) was used to make the results convincing. Take the case of samples from patients with IgA; the technique was applied to a 4-μm-thick kidney section and performed 3D reconstruction of abnormal microvascular. Analysed by the method, vasculars undergoing EndMT were directly-viewed (Fig. 1b). Meanwhile, the percentage of α-SMA+CD31+ peritubular capillary (PTC) numbers were substantially increased in these patients compared with controls (Fig. 1c), suggesting the contribution of endothelial-origin myofibroblasts to interstitial fibrosis in kidney disease.

### Endothelial expression of mesenchymal marker α-SMA is identified in mouse UUO kidney and TGF-β1 treated cells

**Figure 2:**
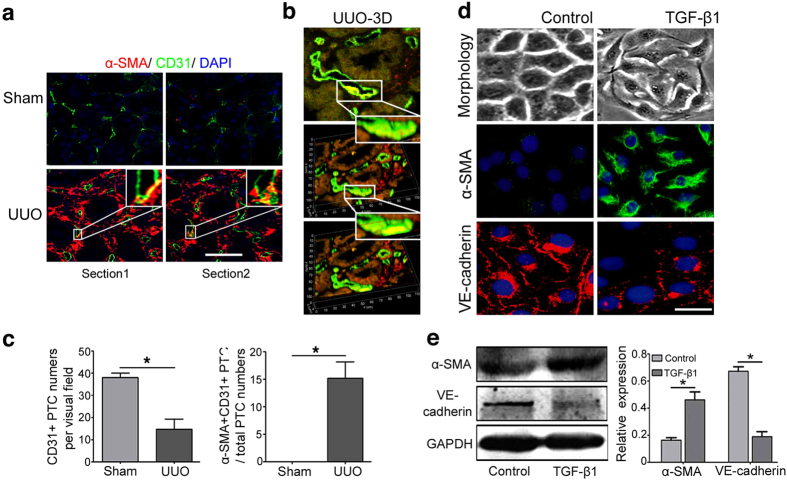
EndMT in the mouse model of UUO and TGF-β1-induced EndMT in HUVECs. (**a**) Serial kidney sections from UUO and sham-operated mice were double stained with antibodies to α-SMA (green) and CD31 (red) at a magnification of ×800 (n = 8, 5 hpf per mouse, 40 hpf per group). (**b**) 3D reconstructed images illustrated microvascular endothelial cells underwent endothelial-to-mesenchymal transition in UUO mice. (**c**) The bar graphs summarized the average of CD31+ PTC numbers per visual field and the percentage of α-SMA+CD31+ PTC numbers in total CD31+ PTC numbers in each group. *P < 0.05. Bars represent means ± sd. (**d**) Phase contrast microscopy showing morphological changes in cells treated with TGF-β1 (10 ng/ml) for 72 h (upper). Cells were double labeled with α-SMA (green) and VE-cadherin (red), and cell nuclei were enhanced by staining with DAPI (middle). (**e**) Lysates were analyzed by western blot for protein expressions of α-SMA and VE-cadherin with densitometry analysis (lower). n ≥ 3. *P < 0.05 versus the control group. Bars represent means ± sd. Scale bar: 20 μm.

To confirm the contribution of EndMT to renal interstitial fibrosis, a mouse model of unilateral ureteral obstruction (UUO) was firstly investigated. Similar to our discovery in patients with IgAN, DN and LN, coexpression of α-SMA and CD31 were substantially increased on serial kidney sections of mice with UUO after 2 weeks of disease induction but rarely observed in sham-operated group (Fig. 2a). 3D reconstructed images illustrated microvascular endothelial cells underwent endothelial-to-mesenchymal transition (Fig. 2b). Next, we established a cell model of EndMT using human umbilical vein endothelial cells (HUVECs) stimulated by TGF-β1, to test our hypothesis that activated fibroblasts can arise from endothelial cells. Compared with basal conditions, certain subsets of endothelial cells exhibited a fibroblast-like morphological phenotype upon stimulation with TGF-β1 for 72 h (Fig. 2d). In cells stained by immunofluorescence with endothelial (VE-cadherin) and mesenchymal marker (α-SMA), we observed loss of VE-cadherin expression and gain of α-SMA in response to TGF-β1 treatment, which was confirmed by analyzing protein expression using western blot (Fig. 2e). These data suggest that endothelial cells can undergo endothelial-to-mesenchymal transition both *in vivo* and *in vitro*.

### PP2A activates in mouse UUO kidney and TGF-β1 treated cells

**Figure 3:**
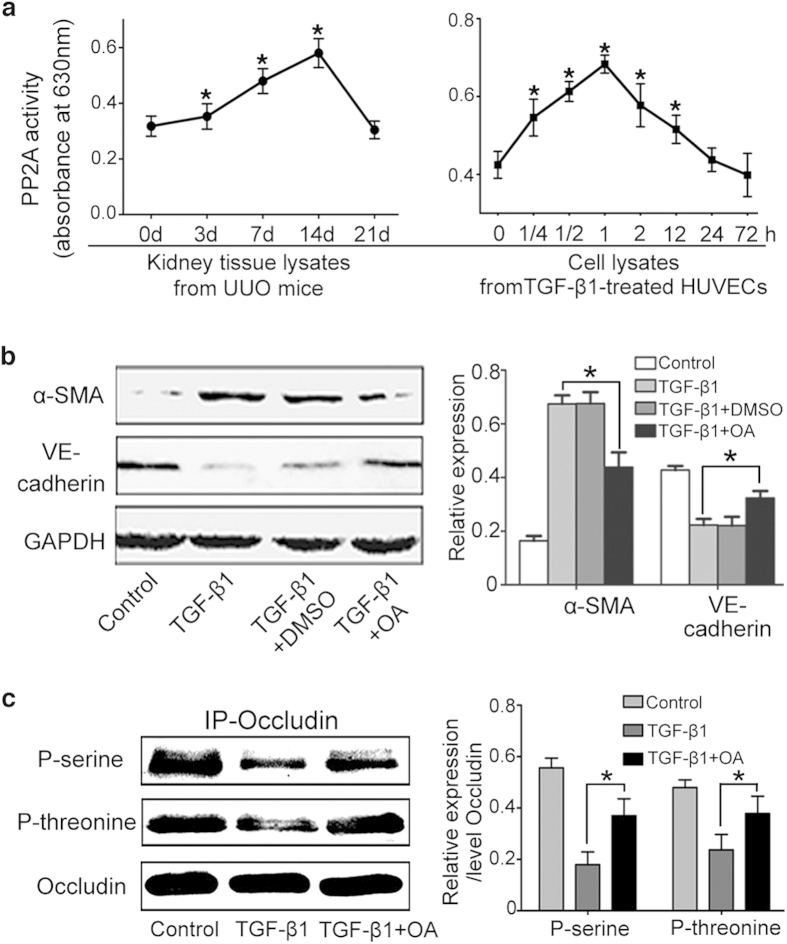
PP2A activates in UUO mice and TGF-β1-treated HUVECs. (**a**) PP2A activity was measured using the Serine/Threonine Phosphatase Assay System in UUO kidney tissues and TGF-β1-treated HUVECs (n = 8). (**b**) OA prevents TGF-β1-induced EndMT. Representative western blot of α-SMA and VE-cadherin in each group with densitometry analysis of protein expression (n ≥ 3). (**c**) OA prevents TGF-β1-induced dephosphorylation of occludin. Cell lysates were subjected to immunoprecipitation (IP) with an anti-occludin anti-body, followed by immunoblotting (IB) with anti-p-threonine and anti-p-serine antibodies. The bargraph shows the average volume density normalized to occludin (n ≥ 3). *P < 0.05 versus the basal condition. Bars represent means ± sd.

As one of the most abundant serine-threonine phosphatases, PP2A accounts for a substantial part of the total phosphatase activity. Therefore, we evaluated PP2A activity both in tissue lysates from UUO mice and cell lysates from TGF-β-treated HUVECs. Notably, PP2A activity was gradually upregulated, nearly twofold in UUO mice of 2 weeks compared with sham-operated group and significantly decreased in UUO mice of 3 weeks. Our data also showed that exposure to TGF-β1 increased PP2A activity in HUVECs, which began at 15 min and peaked at 60 min, a statistically 62% increase compared with the control (Fig. 3a).

To investigate the role of PP2A on EndMT, we cultured HUVECs in the presence of okadaic acid (OA), a specific inhibitor of PP2A phosphatase activity, prior to TGF-β1 treatment. Exposure to OA markedly attenuated α-SMA expression induced by TGF-β1 and maintained VE-cadherin expression (Fig. 3b), indicating PP2A promoted EndMT. To strengthen the results, we analyzed phosphorylation status of PP2A physiological substrates-occludin, an integral membrane protein of tight junctions. TGF-β1 decreased the abundance of phosphorylated serine and threonine residues in occludin immunoprecipitates, which was significantly dampened by pretreatment with OA (Fig. 3c). Taken together, these results indicate that PP2A activates in the process of EndMT and blockade of PP2A signaling can inhibit this process.

### PP2A activation depends on PP2Ac tyrosine nitration during EndMT

**Figure 4:**
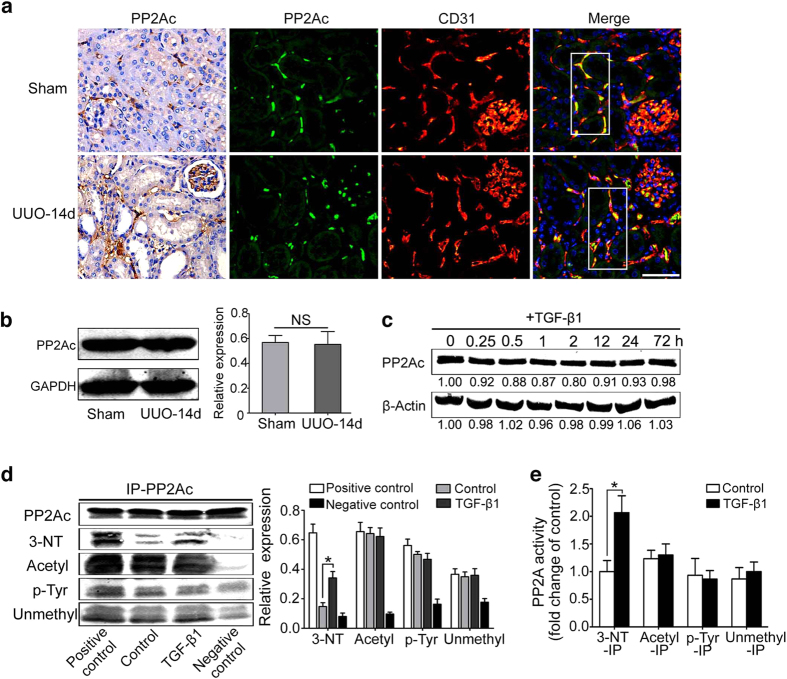
PP2A activation depends on PP2Ac tyrosine nitration during EndMT. (**a**) Representative immunohistochemical detection of PP2Ac in the kidney tissue of sham-operated and UUO mice (left). Co-localization of PP2Ac (green) with CD31(red) was visualized as yellow in the merged images from sham-operated and UUO-14d mice (n = 8). Scale bar: 20 μm. (**b,c**) Western blot analysis of PP2Ac expression in sham-operated and UUO-14d mice (n = 8) and in TGF-β1-treated HUVECs for the indicated periods with densitometry analysis (n ≥ 3). (**d**) HUVECs were incubated with or without TGF-β1 for 72 h and were harvested for immunoprecipitation analysis. Cell lysates were subjected to immunoprecipitation (IP) with an anti-PP2Ac anti-body, followed by immunoblotting (IB) with anti-3-nitrotyrosine (3-NT), anti-acetylation (acetyl), anti-phosphotyrosine (p-Tyr), and anti-methylation (unmethylated) antibodies, respectively. Densitometry analysis of 3-NT, acetyl, p-Tyr and methyl of PP2Ac in d. (**e**) Analysis of PP2A activity in immunoprecipitation of 3-NT-IP, Acetyl-IP, p-Tyr-IP and Unmethylated-IP, expressed as percentage of the value in control group, respectively (n ≥ 3). *P < 0.05 versus the basal condition. NS: no significance. Bars represent means ± sd.

Because PP2Ac is the catalytic subunit of PP2A, we focused on the role of PP2Ac in the induction of EndMT. First, PP2Ac distribution and expression were examined to determine whether there was a correlation between PP2Ac and endothelial cells. Immunohistochemical staining showed that PP2Ac was expressed in the position of endothelium both in glomerular capillaries and interstitial microvasculars of obstructed kidney, without staining in the renal tubules (Fig. 4a). Based on the findings, we assumed that PP2Ac was highly expressed in ECs. To prove the hypothesis, immunofluorescence analysis of double stainings for PP2Ac and CD31 was used. Our results demonstrated that PP2Ac was mainly expressed in renal microvascular endothelial cells (Fig. 4a). No significance was observed in PP2Ac expression between sham-operated mice and UUO mice (Fig. 4b), which was similar to the result from HUVECs for the indicated periods after TGF-β1 treatment (Fig. 4c).

To clarify the role of PP2Ac in the regulation of PP2A, we examined whether PP2Ac post-translational modifications (PTMs) are involved in the activation of PP2A because accumulating evidence demonstrated that the activity and localization of proteins can be regulated by reversible post-translational modifications (PTMs)^18^. We demonstrated that TGF-β1 markedly enhanced the level of 3-nitrotyrosine (a marker of nitration) in PP2Ac using immunoprecipitation assays. A small decrease in phosphorylation was observed, but no modifications in acetylation or methylation were detected (Fig. 4d). Moreover, 3-nitrotyrosine immunoprecipitates isolated from TGF-β1-stimulated endothelial cells also exhibited moderately increased PP2A activity (Fig. 4e), suggesting that PP2Ac nitration is a critical mechanism that activates PP2A.

### Tyrosine 127 (Tyr127) is critical for PP2Ac nitration

**Figure 5:**
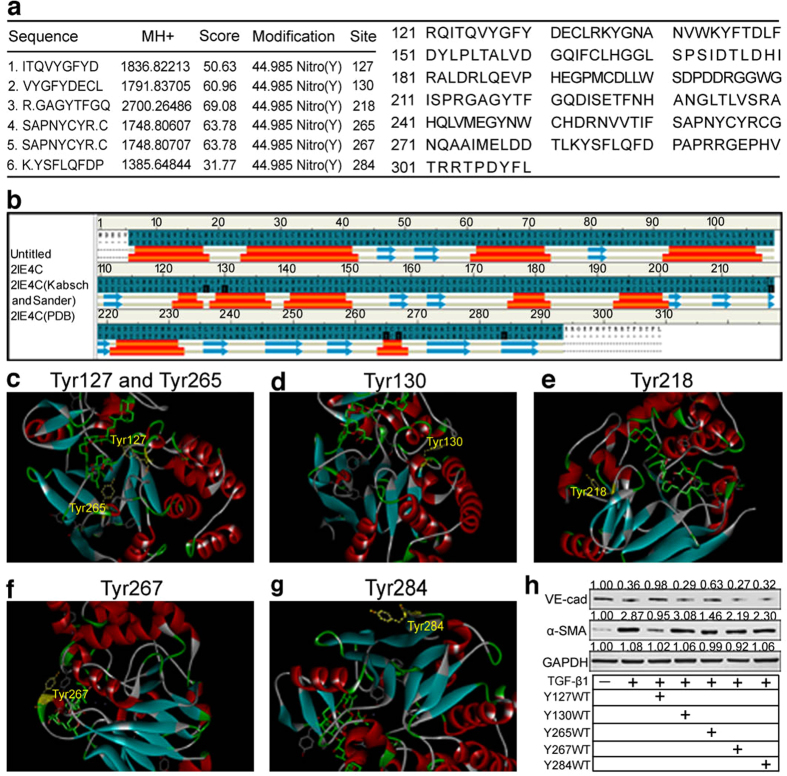
Tyr-127 is critical for PP2Ac catalytic function. (**a**) LC-ESI tandem MS (MS/MS) analysis by Q Exactive detected six peptides sequenced in the spectra. Summary of the PP2Ac modification sites (left). The amino acid sequence is based on NCBI accession number (NP_002706.1) for the mature PP2Acα protein. The numbering of all amino acid residues cited in this report refers to this amino acid sequence of the mature protein (right). (**b**) The figure is based on the secondary structure, deposited as PDB file (PDB accession code: 2IE4C). The helixes are colored orange, and the β-strands and loop switches are colored blue and grey, respectively. (**c–g**) Ribbon diagram of PP2Ac, with residues identified for PP2Ac nitration shown in yellow. (**h**) HUVECs were treated with TGF-β1, TGF-β1 + TAT-Y127WT/Y130WT/Y265WT Y267WT/Y284WT and cultured as untreated controls for 72 h. Representative western blot of VE-cadherin and α-SMA in each group with densitometry analysis (n ≥ 3).

Nitration can be focused on specific tyrosines and potentially result in loss or gain of protein function^19^. Thus, a mass spectrometry assay was performed using recombinant human PP2Ac treated with peroxynitrite to determine targeted residues on PP2Ac. Approximately 1589 CID spectra were recorded. We focused on the amino acid sequence of PP2Ac and used a tyrosine residue mass increase of 45 Da to identify the nitrated spectra. Six peptides containing nitrotyrosine residues (Tyr-127, 130, 218, 265, 267 and 284) were detected in PP2Ac treated with peroxynitrite (Fig. 5a and Fig. S1). In contrast, only Tyr-218 was detected in untreated group. Extensive tests based on PP2Ac structure (PDB accession code: 2IE4C) led to the identification of critical residues in maintaining PP2A phosphatases activity (Fig. 5b). Bioinformatics analysis using ZDOCK 3.0.2 software showed that Tyr127 exhibited higher probabilities for inhibition of OKA (PP2A ligand) binding to the active-site pocket of PP2Ac than the other tyrosines if nitrated (Fig. 5c–g).

To further confirm the hypothesis, PP2Ac mimic peptides (Y127WT,Y130WT,Y265WT, Y267WT,Y284WT) were designed to assess the significance of Tyr127 and other Tyr sites on TGF-β1-driven EndMT. To enhance the penetration of synthetic peptides, a cell-permeable peptide transactivator of transcription (TAT) was used^20^,^21^. The conjugated peptide TAT-Y127WT was labeled with fluorescein isothiocyanate (FITC) and analyzed for cellular uptake. Approximately 5–10 μM peptides exhibited efficient cell penetration and low cytotoxicity to HUVECs (Fig. S2). Thus, at the dose of 10 μM, we investigated the anti-EndMT effects of TAT-Y127WT compared with the other four peptides. As shown in Fig. 5h, Tyr127 exhibited higher potency to inhibit TGF- β1-induced VE-cadherin decrease and α-SMA increase. By analysis their effects on protein expression, an order of inhibitory potency was evaluated: Y127 ≥ 265 > 130 > 284 > 267, indicating that nitrating agents may give priority to Tyr127 to be nitrated and result in the restoration of PP2A activity.

### TAT-Y127WT inhibits EndMT *in vitro*

**Figure 6:**
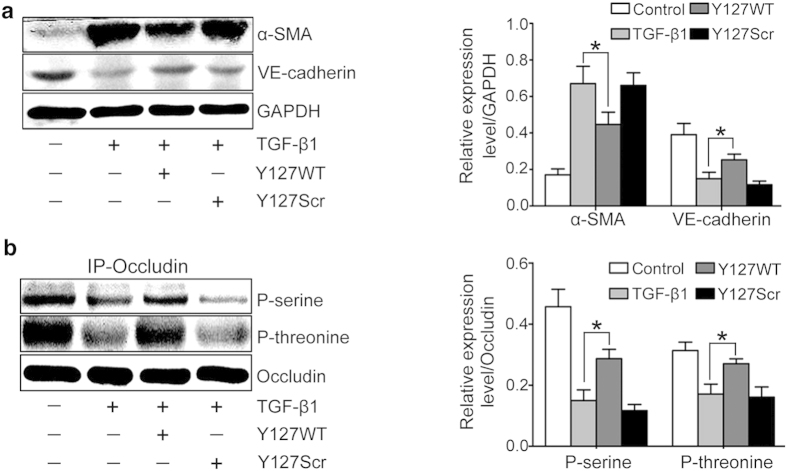
The effect of TAT-Y127WT on EndMT *in vitro*. HUVECs were treated with TGF-β1, TGF-β1+TAT-Y127WT, or TGF-β1+TAT-Y127Scr or were cultured as untreated controls for 72 h. (**a**) Representative western blot of VE-cadherin and α-SMA in each group with densitometry analysis of protein expression (n ≥ 3). (**b**) P-serine and p-threonine levels were measured in occludin immunoprecipitates by western blot analysis. Bar graphs present the densitometric analysis of p-serine and p-threonine (n ≥ 3). *P < 0.05 versus the TGF-β1 group. Bars represent means ± sd.

To further confirm the inhibitory effect of Y127WT on EndMT, PP2Ac Y127WT peptide and its scrambled type (TAT-Y127Scr:TYGVIYQFD) were designed. Pretreatment withTAT-Y127WT matained the expression of endothelial marker VE-cadherin and inhibited the increase of α-SMA (Fig. 6a). Meanwhile, HUVECs with TAT-Y127WT significantly increased both serine and threonine phosphorylation of occludin compared with TAT-Y127Scr treatment (Fig. 6b). In conclusion, TAT-Y127WT blocks TGF-β1-induced cell damage and the EndMT response.

### TAT-Y127WT maintains peritubular capillary density by inhibiting EndMT level and exerts an anti-fibrotic effect *in vivo*

**Figure 7:**
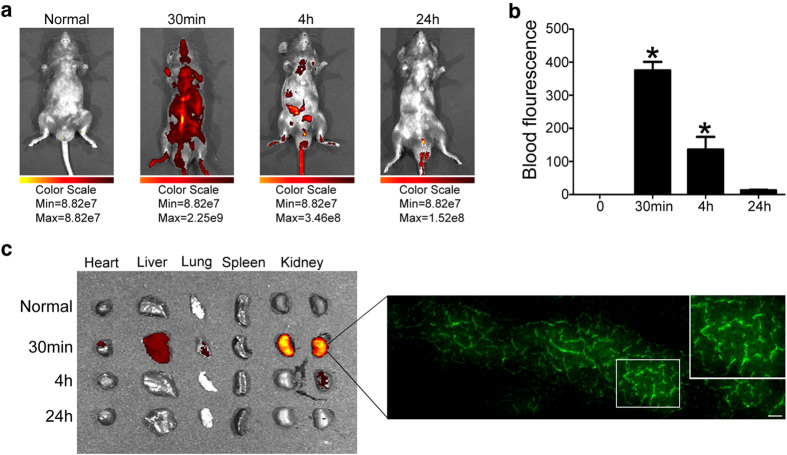
The pharmacokinetics and distribution of TAT-Y127WT *in vivo*. (**a**) Mice were intravenously received 5nmol/g FITC-conjugated TAT-Y127WT. Representative whole-body fluorescence imaging was performed at 0, 0.5, 4 and 24 h post-injection. (**b**) Blood samples were used to detect peptide fluorescence in different time points (n = 6). (**c**) Mice were anesthetized at different time points and the heart, liver, lung, spleen and kidneys were removed and examined for fluorescence with whole body imaging. *P < 0.05 versus the basal condition. Bars represent means ± sd. Scale bar: 50 μm.

TAT-Y127WT showed an inhibitory effect on EndMT, therefore, we examined whether the drug could also protect endothelium undergoing EndMT in renal peritubular capillaries (PTCs) in UUO mice. First, whole-body fluorescence imaging was used to detect the *in vivo* distribution and degeneration of TAT-Y127WT (FITC labeled). Generally, the fluorescence signal disappeared within 24 h after injection (Fig. 7a,b). Organs and tissues were removed at different time points after tail vain injection. Fluorescence was strongly detected in the kidney and liver, with a small signal in the heart and lung but no signal in spleen (Fig. 7c).

We then administered the peptide at a dose of 5 nmol/g per day to UUO mice until the end of the experiments. No adverse side effects were observed with the treatment.

**Figure 8:**
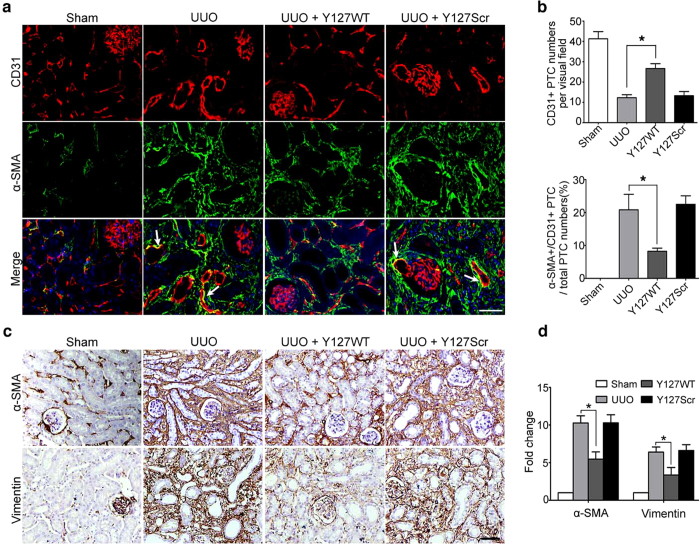
TAT-Y127WT maintains peritubular capillary density by inhibiting EndMT and exerts an anti-fibrotic effect in the UUO-induced renal fibrosis model. (**a**) Colocalization of CD31 (red) with α-SMA (green)is visualized as yellow in the merged images and is marked by white arrowheads (n = 8). (**b**) The bar graphs summarized the average of CD31+ PTC numbers per visual field and the percentage of α-SMA+CD31+ PTC numbers in total CD31+ PTC numbers in each group at a magnification of ×800 (n = 8, 40 hpf per group). *P < 0.05 versus UUO group. Bars represent means ± sd. (**c**) Immunohistochemical staining for α-SMA and vimentin in the obstructed kidneys. (**d**) Fold change of α-SMA+ and vimentin+ areas in each group (n = 8). *P < 0.05 versus the UUO group. Bars represent means ± sd. Scale bar: 50 μm.

Confocal microscopy revealed that the number of CD31+/α-SMA+ areas in the interstitium were dramatically decreased after treatment with TAT-Y127WT (Fig. 8a). Notably, decreased EndMT level was parallel to the preserved density of peritubular capillaries in TAT-Y127WT group (Fig. 8a,b), indicating that TAT-Y127WT successfully inhibits EndMT and promotes PTC recovery.

Given the fact that TAT-Y127WT blocks EndMT and promotes PTC recovery, we investigates whether TAT-127WT has suppressive functions of renal fibrosis. We showed that TAT-Y127WT administration attenuated excessive extracellular matrix (ECM) production in UUO mice, which was measured by α-SMA and vimentin deposition in immunohistochemical staining (Fig. 8c,d). Excessive ECM deposition is the main indicator of renal fibrosis. Thus, TAT-Y127WT showed an inhibitory effect on ECM production, indicating that it is a therapeutic approach in attenuation of renal fibrosis.

## Discussion

In this study, we identified a critical role for PP2Ac Tyr127 nitration in the induction of EndMT via tight junction disassembly. Inhibiting Tyr127 nitration by a novel PP2Ac mimic peptide, TAT-Y127WT, can prevent endothelial cells undergoing EndMT and promotes peritubular capillary recovery in renal fibrosis.

Myofibroblasts play a major role in the synthesis and secretion of extracellular matrix in organ fibrosis, but their precise origin(s) is largely unknown. Although a common notion is that activated fibroblasts arise primarily from resident fibroblasts, differentiation from bone marrow and trans-differentiation from resident quiescent cells are also implicated as important sources of activated fibroblasts^22^,^23^,^24^,^25^. Among types of cell trans-differentiation, endothelial-to-mesenchymal transition was proven to head the list^26^. This process, called EndMT, has been demonstrated to be involved in the progression of tissue fibrosis, including renal fibrosis^27^. In this study, we observed that EndMT occurred in patients with primary and secondary nephropathy (Fig. 1). When we quantified the CD31+/α-SMA+ECs in UUO mice, we found that 10–15% of the total CD31+ cells observed in UUO mice were α-SMA+ (Fig. 2b), which was consistent with the study by Valerie S LeBleu, who suggested that ~10% of vessel-associated endothelial cells contributed to the accumulation of myofibroblasts in fibrotic kidneys subjected to UUO^26^. Therefore, effective targeting of EndMT is essential to prevent renal interstitial fibrosis^27^.

Several signaling pathways were highly focused as potential pathways involving myofibroblasts differentiation and proliferation, including mitogen-activated protein kinase(MAPK)^28^, insulin-like growth factor-1(IFG-1)^29^, integrin pathways^30^, and the classical transforming growth factor-beta 1 (TGF-β)^31^. Meanwhile, the cross-talk between TGF-β/Smad and non-Smad signaling has also made a concerted contribution to myofibroblast activation^32^. There is evidence that TGF-β signaling participates in activated fibroblasts raised from endothelial cells. Partial ablation of TβRII in the endothelium reduced EndMT and fibrosis in chronic kidney disease^33^.Blockade of the TGF-β1/Smad3 signaling pathway could inhibit TGF-β1-induced EndMT^8^. Meanwhile, different sources of myofibroblasts have a reciprocal action for each other. As mentioned above, about 35% of α-SMA+ myofibroblasts were bone marrow derived, which migrated to injured kidney and secreted massive extracellular matrix (ECM), contributing to renal fibrosis^34^. As a consequence, peritubular capillaries in the interstitium can be compressed, resulting in chronic ischemia and hypoxia that could contribute to endothelial dysfunction. ECs dysfunction and the subsequent regression of the capillary network may contribute to the development of the renal interstitial injury, which then may, in turn, lead to locally elevated synthesis and release of chemokines, mediating the recruitment of bone marrow-derived fibroblast precursors into the kidney.

The significance of TGF-β/Smad signaling has been intensively investigated in kidney disease for decades; however, the effects of other signalings remain poorly understood. Herein we focused on the role of PP2A signaling in the activation of fibroblasts in present study. PP2A accounts for as much as 1% of total cellular proteins and represents the major portion of serine/threonine phosphatase activity in most tissue and cells^10^,^35^. PP2A exhibits phosphatase activity by interacting with a substantial number of other cellular proteins, which are PP2A substrates. Occludin is a substrate of PP2A. As an integral membrane protein of tight junctions (TJs), the integrity of occludin depends on endogenous protein phosphatases^36^. Parimal Sheth *et al.* demonstrated that PP2A translocation and dephosphorylation of occludin on threonine residues led to disruption of TJs^37^,^38^. Blocking PP2A prevented the hypoxia-induced occludin reduction at the plasma membrane in alveolar epithelial cells^39^. Indeed, disruption of TJ is an essential step of ECs contributing to fibrosis. Firstly, the loss of TJ impaires ECs adhesions. Consequently, ECs can migrate to the surrounding tissue and be easily stimulated by profibrotic cytokines ultimately becoming myofibroblasts and promoting fibrosis. Secondly, disruption of TJ-induced EndMT results in loss of peritubular capillaries, which would be expected to result in chronic ischemia and hypoxia that could stimulate the scarring process.

Thus it can be seen that interfering with PP2A at the EC level to prevent TJ disruption is a promising strategy for improving renal fibrosis.

During EndMT, ECs lose endothelial cell phenotype and acquire mesenchymal characteristics, including spindle cell morphology, reduction of VE-cadherin expression and induction of α-SMA expression. Of note, the phenotype of cells undergoing transition may contain both endothelial and mesenchymal (myofibroblast) properties. Thus, although a portion of ECs underwent EndMT after 14-d UUO, PP2Ac is not going to decrease because ECs still contain their original property. Therefore, PP2Ac still exists in ECs. The total amount of PP2Ac does not change (Fig. 4b,c).

PP2A dephosphorylation of occludin requires a self-activation, but the exact mechanism is currently unclear. Previous studies demonstrated that PP2A activity and function were regulated by post-translational modifications (PTMs) of PP2Ac amino acid residues^40^,^41^. In the current study, we compared PP2Ac methylation, acetylation, phosphorylation and nitration and their roles in changing PP2A activity. Under TGF-β1 stimulation, PP2Ac underwent significant nitration compared with the other modifications (Fig. 4d). Moreover, 3-nitrotyrosine immunoprecipitates also exhibited moderately increased PP2A activity (Fig. 4e). Indeed, the PTMs of residues directly antagonize other post-translational modifications^42^. Formation of 3-nitrotyrosine in protein tyrosines can inhibit tyrosine phosphorylation, resulting in the activation of the enzyme^43^,^44^. Therefore, we could imagine that the outcome of competing modifications by nitration increases PP2A activity directly or indirectly via specific residue nitration that functions antagonistically with the initial or other modifications.

PP2Ac includes 16 tyrosine residues out of a total of 309 amino acids. To determine the nitration site of PP2Ac, we analyzed the reaction of purified PP2Ac with peroxynitrite. Mass spectrometry revealed that Y127, Y130, Y218, Y265, Y267 and Y284 were nitrated (Fig. 5a). Our findings are compatible with the observations of Takashi Ohama *et al.*, who demonstrated that Tyr284 was nitrated in PP2Ac^45^. To test which nitration site is important for PP2Ac activation, the known crystal structure of PP2Ac was functionally modeled. Y127 locates to the active-site pocket of PP2Ac occupied by OKA under basal conditions. However, if the site undergoes modifications such as nitration, binding of OKA to PP2Ac would be prevented, resulting in the release of PP2A activity^46^. Several factors could also promote Tyr127 selectivity: (1) Y127 is exposed on the protein-chain surface, and this position may be critical for nitration and have a higher probability of being nitrated^47^, (2) known sites of tyrosine nitration appear to be in close proximity to acidic residues^48^. Tyrosine 127 in PP2Ac neighbors two acidic amino acids, aspartic acid and glutamate, within 4–5 AA of Tyr127. These two negative charges also influence the local concentration of the nitrating agent for directing nitration to Tyr127^49^, (3) the side chain of Tyr-127 is in van der Waal’s contact with the active site residues His-118 and Asp-88, which are critical for PP2Ac catalytic function^50^. Together, these factors indicate that Tyr127 fits structural requirements and explains that the Tyr127 site is critical for PP2A activation.

Therefore, blocking PP2Ac Tyr127 nitration is considered a potential therapeutic approach. Recently, a synthetic peptide with the sequence TPDYFL at the C-terminal in PP2Ac was demonstrated to attenuate PP2A translocation in cardiac myocytes^51^. This sequence also prevented acetaldehyde-induced tight junction disruption and barrier dysfunction in Caco-2 Cells^37^. Peptide-based drugs have garnered much attention because of their high potencies of action and relatively few off-target side-effects^52^,^53^. Here, the newly designed peptide TAT-Y127WT effectively inhibits PP2Ac-mediated response as a competitive mimic peptide against Tyr127 nitration. Importantly, administration of TAT-Y127WT to UUO mice significantly reduces PP2Ac nitration and attenuates the expression of key components involved in renal fibrosis, including ECM deposition and capillary rarefaction (Fig. 8). On the basis of these findings, a model is proposed for TAT-Y127WT therapeutic effect, where upon competitive binding of nitrating species to Tyr127, PP2A activation inhibits first, followed by maintaining downstream-substrates phosphorylation status in tight junctions. Finally, cellular integrity and endothelial function are protected.

TAT-Y127WT may be limited due to its lack of cell type-specific targeting, although it has a stable distribution in kidney (Fig. 7c). Due to the scope of the current studies, we have not looked into potential side effects of TAT-Y127WT that could lead to possible injuries in tissue and organs, although our strategy consisting of injections at a low concentration should minimize the risk. Furthermore, TAT itself has been considered a candidate for several medical applications^54^. We need to re-evaluate TAT as a delivery tool with no other known biological functions.

In conclusion, our data reveal a key role for PP2Ac Tyr127 nitration in the regulation of EndMT after renal injury. With the addition of a cell-penetrating motif, TAT-Y127WT successfully suppresses PP2Ac nitration-induced PP2A activtion, thereby inhibiting the loss of endothelial integrity and differentiation into scar-forming myofibroblasts. From these results, we conclude that TAT-Y127WT is promising as a high-performance peptide-based drug toward renal fibrosis.

## Methods

### Subjects

All patients were diagnosed by one pathologist and met the diagnostic criteria for IgA nephropathy (IgAN), diabetic nephropathy (DN) and lupus nephritis (LN). 8 adult patients were recruited to this study in each group. Negative controls were from those patients who underwent renal trauma and required nephrectomy with no history of renal disease. The study was carried out in accordance with the ethical standards of the Helsinki Declaration and approved by the Institution Review Board of of Huazhong University of Science and Technology, Tongji Hospital. Written informed consent was obtained from each patient before any study-specific investigation was performed.

### Cell Culture and Treatment

Human umbilical vein endothelial cells (HUVECs, ATCC) were cultured in RPMI-1640 (Gibco) supplemented with 10% fetal bovine serum (FBS, HyClone). When 30% confluent, cells were cultured in serum-free medium before recombinant human TGF-β1 (10 ng/ml; Peprotech) treatment. In some experiments, cells were pretreated with PP2A inhibitor OA (10 nM; Sigma) for 1 h before the addition of TGF-β1. For all cell stimulations, n ≥ 3 independent experiments were performed.

### Cell Labeling

HUVECs grown on cover slips in 12-well plates were incubated overnight at 4 °C with the indicated primary antibodies followed by incubation with a FITC-or CY3-conjugated antibody (Jackson ImmunoResearch) for 45 min at 37 °C. Subsequently, slides were counterstained with DAPI (DAKO) to visualize the cell nuclei and analyzed by confocal laser scanning microscopy.

### Western Blot Analyses

Renal tissues or cells were harvested and centrifuged at 12,000 g to remove cell debris. Protein concentration was measured using a BCA assay kit (Pierce). Eighty micrograms of protein was subjected to SDS-PAGE and transferred onto polyvinylidene difluoride membranes. After transfer, the membranes were blocked and blotted routinely with antibodies against VE-cadherin (BD Biosciences), α-SMA (Abcam), collagen1 (Abcam), PP2Ac (Cell Signaling Technology), occludin (Invitrogen), P-threonine/P-serine (Santa Cruz), nitrotyrosine (Cayman), acetylated-lysine (Cell Signaling Technology), phosphotyrosine (PY-20,Biolgend), PP2A,C subunit, demethylated (Millipore), vimentin (Boster) and CD31 (Proteintech). The immunolabeled proteins were detected by enhanced chemiluminescence (Pierce).The density of the bands was analyzed by Quantity One software (Bio-Rad).

### Immunoprecipitation

Cell lysates were centrifuged at 12,000g for 30 min at 4 °C. Approximately 1 mg of each lysate was incubated overnight at 4 °C with 2 μg of anti-occludin, anti-PP2Ac or IgG antibody (a negative control), followed by incubation with Protein A/G-Agarose for 1 hr to capture immunoprecipitates. Bead complexes were washed four times in lysis buffer and then resuspended in 2 × SDS buffer and heated for 10 min at 90 °C. Lysates were electrophoresed, transferred to PVDF filters, and subsequently immunoblotted with antibodies.

### PP2A activity

Protein Ser/Thr phosphatase activity was assayed photometrically using the Serine/Threonine Phosphatase Assay System (Promega) according to the manufacturer’s directions.

### Mass Spectrometry Experiments

Detailed methods are provided in the supplementary material.

### Bioinformatics analysis

ZDOCK 3.0.2 software was used to predict the structure of a complex between PP2Ac and its ligand OKA. The ZRANK program was used for docking refinement and providing accurate rescoring of models from initial-stage docking^55^.

### Peptides

Y127WT (ITQVYGFYD) and its randomly scrambled variant peptide Y127Scr (TYGVIYQFD) were synthesized. Meanwhile, Y130WT, Y265WT, Y267WT and Y284WT were synthesized. Fluorescein iso-thiocyanate (FITC) was coupled to the peptides via an additional lysine at the N-terminus in whole-body imaging experiments. The peptides were synthesized using fluorenylmethyloxy carbonyl chloride chemistry in a solid-phase synthesizer and purified by high-performance liquid chromatography, and their sequences and structures were confirmed by mass spectrometry (Wuhan Bai yixin Bio-tech Ltd., Wuhan, China).

### *In Vivo* Imaging

FITC-loaded peptides were injected through the tail vein or intraperitoneally administered into C57/B mice at a dose of 5 nmol/g of body weight. Whole body fluorescence imaging was performed using an *in vivo* imaging system (IVIS Spectrum, Caliper, USA) at 0, 0.5, 4 or 24 h post-injection. Subsequently, the mice were sacrificed, and their organs such as the liver, spleen, kidneys, heart, and lung were excised and subjected to *ex vivo* fluorescence imaging. At the same time, 1 mL of blood was drawn from the retro-orbital sinus after injection at different time points.

### UUO Animal Model

Mice aged 6–8 weeks were grouped as sham-operated, UUO, UUO +TAT-Y127WT and UUO + TAT-Y127Scr and tail vein injected 1d before surgery. To perform UUO surgery, mice were anesthetized with ketamine hydrochloride (80 mg/kg body weight, intraperitoneally) before surgery and the left kidney was exposed through a flank incision and the left ureter tied off at the level of the lower pole of the kidney with two 4.0 silk ties. All mice were euthanized at the end of the experiments after obstruction, and some kidney tissues were fixed with 4% paraformaldehyde. The remainder was frozen in liquid nitrogen for later use. All animal studies were performed in accordance with our university’s guidelines for animal care.

### Immunohistochemistry and Immunofluorescence

Four-micrometer-thick paraffin sections were incubated with primary antibodies overnight at 4 °C and were then incubated with biotinylated goat anti-rabbit IgG or goat anti-mouse IgG for 45 min followed by diaminobenzidine (DAKO) staining. All sections were examined under a light microscope (Olympus BX-50) and digitized with a high-resolution camera. For immunofluorescence studies, after incubated with primary antibody, sections (including serial sections) were incubated with FITC- or CY3-conjugated goat antibody (Jackson ImmunoResearch) for 45 min at 37 °C and then counterstained with DAPI (Vector Laboratories) and analyzed by confocal laser scanning microscopy. 3D reconstructed technology was based on confocal laser-scanning microscopy and analyzed by the software (LAS-AF-Lite 2.6.3). PTC density was analyzed as described with some modifications^56^.

### Statistical Analyses

Data were analyzed using standard statistical methods, including linear regression, the *t* test and one-way ANOVA using SPSS (version 15.0). Data are expressed as the mean ± sd. P < 0.05 was considered statistically significant.

## Additional Information

**How to cite this article**: Deng, Y. *et al.* Blocking protein phosphatase 2A signaling prevents endothelial-to-mesenchymal transition and renal fibrosis: a peptide-based drug therapy. *Sci. Rep.* **6**, 19821; doi: 10.1038/srep19821 (2016).

## Supplementary Material

Supplementary Information
