# Supplementary material for: Blocking protein phosphatase 2A signaling prevents endothelial-to-mesenchymal transition and renal fibrosis: a peptide-based drug therapy
Source: Sci Rep. 2016 Jan 25;6:19821. doi: 10.1038/srep19821 (PMC4726189; doi:10.1038/srep19821)
Supplement: Supplementary Information [file srep19821-s1.pdf]

Blocking protein phosphatase 2A signaling prevents endothelial-to-mesenchymal transition and renal fibrosis: a peptide-based drug therapy

Yuanjun Deng[1], Yanyan Guo[1], Ping Liu[1], Rui Zeng[1], Yong Ning[1], Guangchang Pei[1], Yueqiang Li[1], Meixue Chen[1], Shuiming Guo[1], Xiaoqing Li[1], Min Han[1,*], Gang Xu[1,*]

Supplementary material

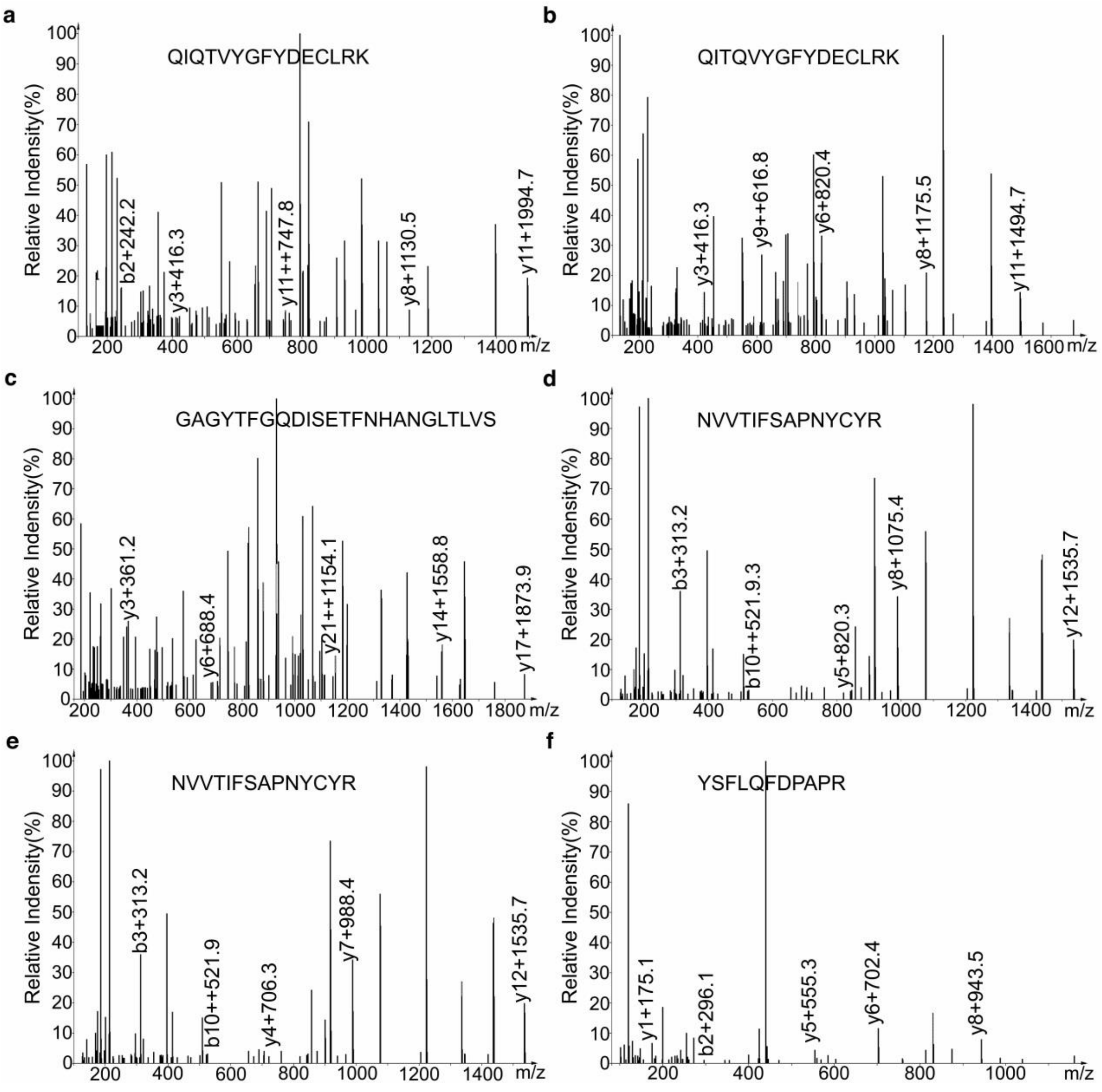

Figure S1. LC-ESI tandem MS (MS/MS) analysis by Q Exactive detected six peptides sequenced in the spectra. The spectra were acquired during the analysis of in-gel tryptic digests of the peroxynitrite-treated PP2Ac band.

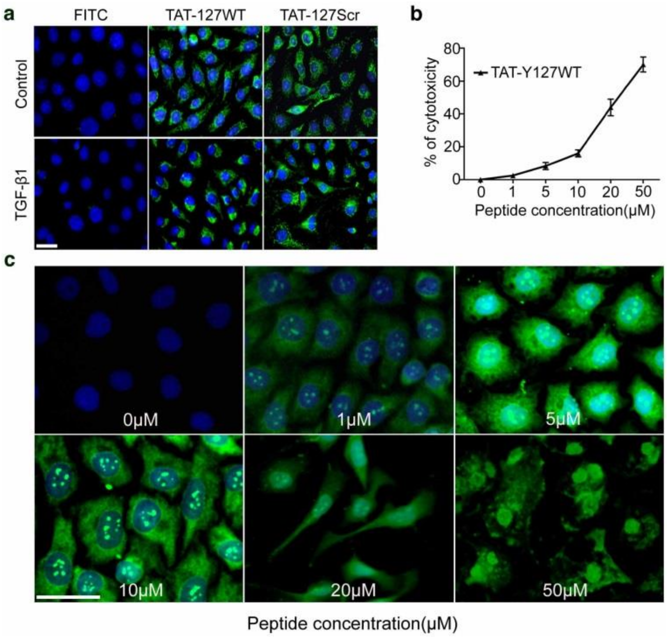

Figure S2. The penetration and cytotoxicity of TAT-Y127WT to HUVECs. (a)TAT-Y127WT effectively transduces cells at a concentration of 10 μM. (b) HUVECs were incubated with various concentrations (0,1,5,10,20,or 50μM) of TAT-Y127WT. Cytotoxicity was measured with the CCK-8 assay. (c) Immunofluorescence distribution of TAT-Y127WT and cell morphology. Nuclei were counterstained with DAPI (blue). n=3. Scale bars, 20μm.

Supplementary Methods: Mass Spectrometry Experiments

Protein Digestion-The protein bands were digested according to an in-gel digestion procedure. The gels were dried in a vacuum centrifuge. Briefly, the in-gel proteins were reduced with dithiothreitol (10 mM DTT/100 mM NH4HCO3) for 30 min at 56 ℃, then alkylated with iodoacetamide (200 mM IAA/100 mM NH4HCO3) in the dark at room temperature for 30 minutes. Gel pieces were briefly rinsed with 100 mM NH4HCO3 and ACN. Gel pieces were digested overnight in 12.5 ng/μl trypsin in 25 mM NH4HCO3. The peptides were extracted three times with 60% ACN/0.1% TFA. The extracts were pooled and dried completely by a vacuum centrifuge.

Identification of the Modification Sites-Experiments were performed on a Q Exactive mass spectrometer that was coupled to Easy nLC (Proxeon Biosystems, now Thermo Fisher Scientific). Approximately 3 μl of the sample was injected for nanoLC-MS/MS analysis. The peptide mixture (1 μg) was loaded onto the C18-reversed phase column

(15 cm long, 75 μm inner diameter) packed in-house with RP-C18 5-μm resin in buffer A (0.1% Formic acid) and separated with a linear gradient of buffer B (80% acetonitrile and 0.1% Formic acid) at a flow rate of 250 nl/min controlled by IntelliFlow technology over 70 min. MS data were dynamically acquired using a data-dependent top10 method by choosing the most abundant precursor ions from the survey scan (300-1800 m/z) for HCD fragmentation. Determination of the target value is based on predictive Automatic Gain Control (pAGC). Dynamic exclusion duration was 25 s. Survey scans were acquired at a resolution of 70,000 at m/z 200, and the resolution for HCD spectra was set to 17,500 at m/z 200. Normalized collision energy was 30 eV, and the underfill ratio, which specifies the minimum percentage of the target value likely to be reached at maximum fill time, was defined as 0.1%. The instrument was run with the peptide recognition mode enabled.

Sequence Database Searching and Data Analysis-MS/MS spectra were searched using MASCOT engine (Matrix Science, London, UK; version 2.2) against Uniprot human database (133549 sequences, download Mar. 3rd, 2013). For protein identification, the following options were used. Peptide mass tolerance=20 ppm, MS/MS tolerance=0.1 Da, Enzyme=Trypsin, Missed cleavage=2, Fixed modification: Carbamidomethyl (C), Variable modification: Oxidation (M), and nitration (W,Y).All reported data were filtered by score=20 and expect value=0.05.
